# Divergent effects of high-intensity functional training and moderate-intensity continuous training in adolescents with overweight/obesity: a randomized controlled trial on body composition, physical fitness, and psychological health

**DOI:** 10.3389/fphys.2026.1756285

**Published:** 2026-03-16

**Authors:** Junwei Liu, Yanlin Zhao, Meng Cao, Zhanglin Chen, Hui Zhou, Tong Ke, Yishan Chen, Changfa Tang

**Affiliations:** 1 National Key Laboratory of Human Movement Simulation and Sports Promotion, College of Physical Education, Hunan Normal University, Changsha, China; 2 School of Tourism, Physical Education and Health, Guilin University, Guilin, China; 3 College of Physical Education and Health, Guangxi Normal University, Guilin, China; 4 Institute of Physical Education, Normal College, Shenzhen University, Shenzhen, China; 5 Faculty of Health and Wellness, City University of Macau, Taipa, Macao SAR, China

**Keywords:** adolescent over-weight/obesity, high-intensity functional training, mental health, moderate-intensity continuous training, mood states, precision intervention

## Abstract

**Objectives:**

In the wake of the COVID-19 pandemic, declines in physical fitness and psychological resilience among adolescents with overweight/obesity have underscored the need for effective and feasible school-based behavioural interventions. This study compared the effects of High-Intensity Functional Training (HIFT) and Moderate-Intensity Continuous Training (MICT) on body composition, physical fitness, and multidimensional mental health in overweight/obese adolescents.

**Methods:**

In this randomized controlled trial, adolescents aged 11–12 years with overweight/obesity were assigned to an 8-week school-based intervention of HIFT, rhythm-/music-accompanied MICT, or a control condition (three sessions per week). Body composition, physical fitness, mood states, and mental health were assessed before and after the intervention. Dietary intake was monitored to ensure stability throughout the study period.

**Results:**

Both HIFT and rhythm-based MICT improved body composition, physical fitness, and psychological outcomes compared with the control group. MICT showed stronger effects in reducing overall adiposity and attenuating negative mood and internalizing symptoms such as anxiety and depressive mood. In contrast, HIFT produced greater improvements in cardiorespiratory fitness, muscular fitness and power, and positive mood dimensions including vigor and self-esteem, and showed additional benefits for interpersonal sensitivity.

**Discussion:**

Under real-world school conditions, both HIFT and rhythm-based MICT improved body composition, fitness, and mental wellbeing in adolescents with overweight/obesity, but with differential response profiles. These findings support a more tailored (“precision-oriented”) selection of school-feasible exercise modalities based on prioritized physical and psychological targets.

**Clinical Trial Registration:**

https://www.chictr.org.cn/showproj.html?proj=61446, identifier ChiCTR2100048737.

## 1 Introduction

Adolescent obesity represents an escalating global public health crisis. According to the WHO, over 390 million children and adolescents aged 5–19 years were overweight in 2022. The prevalence of overweight (including obesity) among children and adolescents aged 5–19 has risen dramatically from just 8% in 1990 to 20% in 2022. The rise has occurred similarly among both boys and girls: in 2022, 19% of girls and 21% of boys were overweight (World Health Organization, 2025). This trend poses substantial health risks that extend beyond established metabolic and cardiovascular consequences (Malone and Hansen, 2019). Particularly concerning is the bidirectional relationship between obesity and declining physical fitness—characterized by impaired cardiorespiratory function and reduced muscular strength—which often creates a self-perpetuating cycle of physical inactivity and health deterioration (King et al., 2012; Chen et al., 2022).

This physical health crisis is further compounded by significant mental health challenges. Adolescents with obesity face substantially elevated risks of negative emotional states, including diminished self-esteem, depression, and anxiety (Halfon et al., 2013). This frequently establishes a detrimental feedback loop wherein psychological distress undermines engagement in health-promoting behaviours, thereby exacerbating both physical and mental health outcomes. The post-pandemic era has intensified these challenges, with extended home confinement and reduced physical activity worsening both obesity rates and psychological distress among youth (Sa Filho et al., 2020; Jones et al., 2021). Adolescence constitutes a critical period for obesity onset and persistence (Alberga et al., 2012b), while simultaneously representing a key window for exercise-mediated health improvement (Alberga et al., 2016).

Schools provide an essential setting for implementing adolescent health interventions (Bogataj et al., 2021). In response to this multifaceted challenge, research has primarily focused on traditional exercise modalities, establishing moderate-intensity continuous training (MICT) as a foundational intervention with demonstrated benefits for metabolic health, body composition (Mendonca et al., 2022) and emotional wellbeing (Xie et al., 2021). However, conventional MICT is frequently limited by monotony, leading to poor adherence among adolescents—a critical barrier to long-term effectiveness in school settings (Liu et al., 2020). To address this engagement gap, contemporary studies have enhanced MICT protocols by incorporating music and rhythmic choreography, showing promising results for improving exercise enjoyment and participation (Peng et al., 2024). Concurrently, high-intensity functional training (HIFT) has emerged as a viable alternative. While grounded in high-intensity interval training principles, HIFT is distinguished by its varied functional movements and typically group-based format, potentially enhancing adherence through intrinsic motivation and social support mechanisms (Heinrich et al., 2015; Sharp et al., 2022; Koon et al., 2023). Preliminary evidence indicates that HIFT may yield superior improvements in certain physical fitness parameters compared to traditional approaches (Heinrich et al., 2012; Heinrich et al., 2015; Murawska-Cialowicz et al., 2015). Adjustable strength design enhances exercise tolerance (Bartlett et al., 2011; Sharp et al., 2022) and is particularly suitable for individuals with a weak exercise foundation (Parfitt et al., 2006).

Despite these advances, existing research still has limitations. First, although both optimized MICT and HIFT aim to enhance engagement, they operate through distinct pathways—MICT via structured enjoyment and rhythmic consistency, versus HIFT through functional variety and social dynamics. A direct comparison of these modalities within identical school-based interventions remains lacking. Second, existing research has emphasized physiological outcomes, with insufficient attention to comparative psychological benefits, particularly regarding domain-specific mental health effects in overweight/obese adolescents. Finally, the understanding of how these exercise modalities differentially influence the physical-psychological health interplay remains limited. Consequently, a critical question emerges: how do these distinct yet engaging exercise modalities compare in addressing the intertwined physiological and psychological health challenges of overweight/obese adolescents?

This study, therefore, aims to systematically compare the effects of rhythm-based, music-accompanied MICT versus group-based HIFT on body composition, physical fitness, and emotional states in overweight/obese adolescents within a school setting. We hypothesize that: (i) Both interventions will improve physical fitness, with HIFT producing greater gains in cardiorespiratory fitness and muscular fitness. (ii) Both interventions will improve mood states, but with different profiles: HIFT will enhance positive mood (e.g., vigor and self-esteem), whereas rhythm-based MICT will reduce negative mood (e.g., fatigue and depressive affect). (iii) The interventions will show differential effects across multidimensional mental health domains, with HIFT preferentially improving interpersonal sensitivity and rhythm-based MICT showing greater reductions in anxiety and depressive symptoms.

## 2 Materials and methods

### 2.1 Study design

The study was conducted from September to November 2022 in a public middle school in China according to the Consolidated Standards of Reporting Trials (CONSORT). Participants were allocated to HIFT, MICT, or CON using a computer-generated random number sequence (SPSS) created by an independent staff member who was not involved in recruitment, training delivery, or outcome assessment. The randomization list was stored in a password-protected file held by the statistician; recruiters and outcome assessors had no access to group assignments. Because the intervention involved supervised exercise, participants and coaches could not be blinded. However, outcome assessors and the statistician/data analyst remained blinded to group allocation throughout testing and analysis, and participants were asked not to disclose their group during assessments. During the intervention period, the students in both intervention groups continued to attend physical education classes as usual. The training sessions were held after class (on Monday and Wednesday afternoons) and during club time (on Friday afternoons). The control group participated in school physical education courses and club courses (unstructured physical activities). All outcome measures were collected at two time points: within the 7 days immediately preceding the start of the intervention (baseline, T0) and within the 7 days following the final intervention session (post-intervention, T1).

### 2.2 Participant characteristics

The study was conducted in the context of an ongoing school fitness-enhancement initiative, which was promoted as improving physical fitness and performance in the school physical examination. Notifications were disseminated to Grade 7 students, and several classes participated as intact classes in the school program. During routine school health screening, height and weight were measured, and body mass index (BMI) was calculated. Students classified as overweight (BMI ≥85th percentile) or obese (BMI ≥95th percentile) based on age- and sex-specific BMI reference standards were privately invited to participate in a “physical fitness enhancement program” aimed at improving physical fitness and performance in the school fitness examination. Weight status was handled confidentially to minimize potential weight stigma. The inclusion criteria were as follows (Alberga et al., 2012a): (1) aged 11–12 years; (2) classified as overweight (BMI ≥85th percentile) or obese (BMI ≥95th percentile); (3) body weight stable (no significant fluctuations) over the preceding 1–2 months; (4) good compliance and ability to complete the testing and training regimen; and (5) no history of surgery within the recent 3 months. The exclusion criteria were (Alberga et al., 2012a): (1) secondary or drug-induced obesity; (2) presence of cardiovascular diseases, diabetes, or dyslipidemia; (3) engagement in regular exercise (≥2 times/week for ≥20 min/session) within 3 months before the study; (4) any contraindications to exercise; (5) unwillingness to participate in exercises at the scheduled times and locations; (6) severe cognitive impairments that could hinder the ability to follow instructions correctly; (7)Participated in the training of other project teams; and (8) unwillingness of the participant or their guardian to provide written informed consent. All participants and their parents provided written informed consent before the study. Before the experiment began, all participants completed the Physical Activity Readiness Questionnaire+ (PAR-Q+) to assess their daily physical activity levels and individual health status (Kessler et al., 2012). This trial was prospectively registered in the Chinese Clinical Trial Registry (ChiCTR2100048737) on 15 July 2021. The registry lists an overall planned implementation and recruitment window (1 September 2021–31 December 2024) to accommodate recruitment across school terms and, where applicable, follow-up assessments. The present manuscript reports the cohort recruited and completing the 8-week intervention between September and November 2022, which falls within the registered timeframe. The trial was approved by the Bioethics Committee of Biomedical Research of Hunan Normal University (Approval No. 2022-391) and conducted in accordance with the Declaration of Helsinki.

The required sample size was calculated *a priori* using G*Power (version 3.1). The calculation assumed α = 0.05, power = 0.80, and an effect size of Cohen’s f = 0.35 (between medium and large according to conventional benchmarks) (Faul et al., 2007). The study design involved three groups (HIFT, MICT, CON) with measurements taken at two time points (pre- and post-intervention), and the analysis indicated that a minimum of 13 participants per group was required.

**FIGURE 1:**
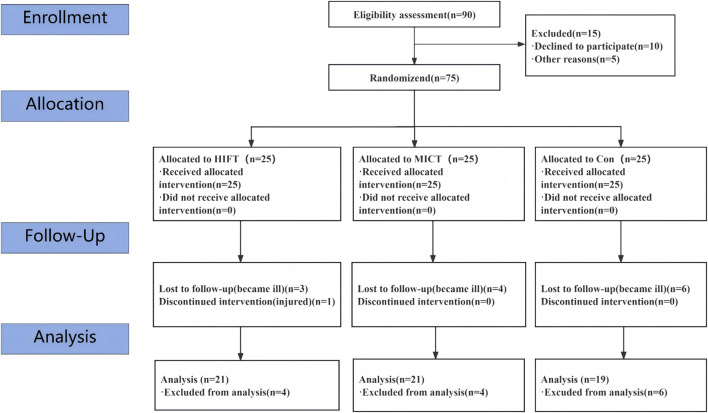
Flow diagram of participant selection.

During routine health screening of Grade 7 students (approximately 16 classes), 90 adolescents meeting the overweight/obesity eligibility criteria were identified and privately invited. Of these, 75 provided written consent and were randomized. Fourteen participants did not complete the post-test assessment due to COVID-19–related absences and minor injuries (Figure 1). Therefore, 61 participants with complete pre- and post-intervention data were included in the final (complete-case) analysis. Baseline characteristics of the analyzed sample are presented in Table 1.

**TABLE 1:** Baseline demographic characteristics of participants included in the final analysis (N = 61).

Indicator	Total (N = 61)	HIFT (n = 21)	MICT (n = 21)	CON (n = 19)	p
Age (years)	11.97 ± 0.18	11.95 ± 0.22	11.95 ± 0.22	12.00 ± 0.00	0.639
Sex (male/female)^a^	25/36	8/13	9/12	8/11	0.945
Height (cm)	1.57 ± 0.07	1.56 ± 0.07	1.57 ± 0.08	1.60 ± 0.07	0.085
Weight (kg)	58.67 ± 7.69	58.01 ± 8.26	56.84 ± 7.84	61.41 ± 6.36	0.153
Body mass index (kg/m^2^)	23.48 ± 1.86	23.76 ± 2.05	23.00 ± 1.59	23.72 ± 1.90	0.322
Overweight/Obesity	42/19	15/6	15/6	12/7	—

HIFT, high-intensity functional training; MICT, moderate-intensity continuous training; CON, control training; p-value refers to the significance of differences between the two groups. a, Chi-square test.

### 2.3 Measurements

Four trained research assistants conducted the measurements. To maximize intra-rater reliability and minimize inter-rater variability, each assistant was assigned to specific, fixed measurement tasks (e.g., one assistant measured all heights and weights, another measured all waist and hip circumferences). Crucially, for each participant, the same assistant performed both the baseline and post-intervention assessments for their assigned tasks. All assistants completed a 1-week centralized training program prior to the study, based on international standard guidelines. A duplicate measurement protocol was used for circumferences: each circumference was measured twice consecutively; if the two readings differed by more than 0.5 cm, a third measurement was taken, and the mean of the two closest values was recorded as the final result. All assessments were conducted at the same time of day (±2 h) for each participant to control for diurnal variation.

#### 2.3.1 Body composition

Body composition, weight, and fat percentage were assessed before and after the intervention using a bioelectrical impedance analyzer (InBody 770, Seoul, South Korea), which was calibrated daily according to the manufacturer’s guidelines. The body composition measurement procedure adhered to a standardized protocol. Consistent with previous studies (McLester et al., 2020; Buch et al., 2022), all measurements were performed before 10 a.m. The participants were asked to fast for at least 8 h before measurements, avoid any intentional physical activity, and wear lightweight clothing on the morning of the examination, when they were measured by standing barefoot on the device platform. To control for confounding variables, the participants were instructed to report their bowel and bladder status (i.e., empty or full) and menstrual cycle phase (i.e., follicular or luteal) before the measurements.

Body mass index (BMI): weight (kg)/height (m^2^). Height was measured using a portable stadiometer (Seca 213, Hamburg, Germany), calibrated before each testing session.

Waist circumference (WC): WC was measured at the midpoint between the lowest rib and the iliac crest using a nonelastic tape (Luoxin, China), recorded to the nearest 0.1 cm.

Hip Circumference (HC): Participants were asked to assume a standardized standing position with feet together, legs extended, and arms hanging naturally. HC was measured using a nonelastic tape measure (Luoxin, China), with the anterior tip positioned at the pubic symphysis and the posterior tip at the point of maximal gluteal protrusion. Three consecutive measurements were taken, and the mean value was reported, rounded to the nearest 0.1 cm. Participants were advised to wear light clothing to mitigate potential interference.

Waist-to-Hip Ratio (WHR): The WHR was derived from the formula WHR = WC/HC.

#### 2.3.2 Cardiorespiratory fitness and blood pressure measurement

Maximum oxygen uptake measurement: The direct measurement of maximal oxygen uptake ( $\dot{V}$ O_2_max is considered the criterion method for assessing cardiorespiratory fitness (Ross et al., 2016). In this study, participants underwent a graded exercise test (GXT) on a treadmill, with respiratory gas exchange analyzed using a stationary metabolic system (MetaLyzer 3B-R2, Cortex Biophysik GmbH, Leipzig, Germany). Pre-test calibration was performed as follows: (1) prior to exercise testing, the cardiorespiratory fitness testing system was calibrated using ambient air, and volume calibration was conducted before daily measurements; if changes in environmental conditions occurred, additional calibration using standard reference gases was performed; (2) during testing, room temperature was maintained at 20 °C–25 °C and relative humidity at 40%–60%. The same protocol was applied to both males and females. Participants were asked to refrain from strenuous exercise for 12 h before testing. Following a 3-min rest period, the test began at a speed of 2.7 km/h and a 2° incline. It progressed in 2.5-min stages, with incremental increases in speed and incline at each stage. Perceived exertion was assessed using the Borg RPE scale (Borg, 1982) during the final 10 s of each stage. Heart rate was recorded continuously throughout the GXT and all training sessions via the Polar Team Pro system (Polar Team Oh1, Polar Electro, Kempele, Finland). Test termination criteria included: (1) achievement of a VO_2_ plateau, (2) respiratory exchange ratio ≥1.10, (3) heart rate reaching ≥90% of the age-predicted maximum (220 − age), and (4) volitional exhaustion leading to test termination (Cunha et al., 2010). Given the time-consuming nature of the $\dot{V}$ O_2_max test, all assessments were scheduled within the 4 days immediately preceding and the 4 days immediately following the intervention period.

Blood pressure measurement: After relaxation in a seated position for approximately 5 min, resting systolic and diastolic blood pressures (SBP and DBP) were measured using an automatic BP monitor (Omron BP652, Omron Healthcare Inc., Vernon Hills, IL, United States).

#### 2.3.3 Physical fitness

Standing Long Jump: This test was employed to measure lower-limb explosive power (Wang et al., 2024). Starting from a position with feet shoulder-width apart behind the starting line, participants executed a maximal-effort horizontal jump using an arm swing for momentum. The jump distance was measured from the start line to the closest point of contact upon landing. Each individual performed three attempts, with the best result retained (recorded in cm to the nearest 0.1 cm).

Handgrip Strength: Handgrip strength was assessed as a proxy for overall musculoskeletal fitness in adolescents (Janz et al., 2021). A digital handgrip dynamometer (Leikang, China) was used for measurement. With the arm held at an angle of approximately 45°, participants applied maximum force for 3 s. The test was administered twice with 30-s rest intervals, and the highest reading was documented in kilograms. Grip strength index: (Grip strength value/Weight) x 100.

Push-ups: Push-up performance was evaluated using gender-specific protocols: males performed standard push-ups (maintaining a straight body line and bending elbows to 90°), while females performed modified kneeling push-ups (with knees supported on a mat and legs elevated at a 45° angle). The test was conducted at a rhythm of 60 beats per minute using a metronome. Termination criteria included two consecutive incorrect repetitions, postural breakdown (uncorrected following two verbal prompts), or self-discontinuation. The total count of correctly executed repetitions was taken as the final score. All sessions were supervised and conducted with safety mats in place.

50-m Sprint Test: This test assessed running speed and anaerobic capacity. Each participant performed a maximal-effort sprint over a 50-m course on a standard running track. Time was measured manually using a stopwatch (Casio, Tokyo, Japan) calibrated to 0.01 s, following standard athletic timing procedures. The elapsed time from the start command to the participant crossing the 50-m finish line was recorded as the result (Fjortoft et al., 2011).

1-Minute Jump Rope Test: The subject assumes a standing position with feet naturally separated and arms extended downward, holding a standard-length jump rope positioned parallel to the ground. Upon the verbal signal, the subject commences continuous jumping. The total number of jumps completed in 1 minute is recorded using a stopwatch, where a valid jump is defined as the rope passing completely beneath both feet. The test is terminated prematurely, and the count at that point is documented if any interruption lasts longer than 3 s.

#### 2.3.4 Mood states

Emotional status was assessed using the Profile of Mood States (POMS) questionnaire. Revised by Bei li Zhu, the simplified Chinese version (Zhu, 1995) includes 40 items across seven dimensions: tension, anger, fatigue, depression, vigor, confusion, and self-esteem. The participants rated their subjective feelings over the past week on a five-point Likert scale. Standardized scoring formulas were used to compute total mood disturbance (TMD), which reflected overall emotional imbalance. The simplified Chinese POMS used in this study has demonstrated good test–retest reliability and construct validity in Chinese children and adolescents, with reported Cronbach’s α coefficients generally 0.71–0.89 (Chi and Lin, 2003) and strong associations with relevant health outcomes (Zhang et al., 2014; Zhang et al., 2020).

#### 2.3.5 Mental health scale

The mental health status of participants was assessed using the Middle School Student Mental Health Scale (MSSMHS), developed by Wang Jisheng specifically for Chinese adolescents in accordance with their cultural characteristics and behavioural patterns (Wang et al., 1997). As a symptom-oriented diagnostic instrument, the MSSMHS is designed to screen for psychological problems or disorders among secondary school students. The scale consists of 60 mandatory items evaluating 10 common psychological domains, including obsessive-compulsive tendencies, paranoid ideation, hostility, interpersonal sensitivity, depression symptoms, anxiety, academic stress, maladjustment, emotional instability, and psychological imbalance. Although the MSSMHS was developed in China, its domain structure aligns conceptually with widely used international symptom-based instruments. For example, the anxiety and depression subscales correspond to core internalizing symptom dimensions assessed in tools such as the SCL-90 and RCADS; interpersonal sensitivity reflects social evaluative concerns and peer-related distress; obsessive-compulsive tendencies parallel compulsive cognition and behavior dimensions; and emotional instability overlaps with affective dysregulation constructs. The inclusion of domains such as academic stress and maladjustment further captures culturally salient stressors relevant to Chinese adolescents. Therefore, the MSSMHS provides both cultural specificity and conceptual comparability with internationally recognized mental health constructs, making it an appropriate instrument for evaluating multidimensional psychological outcomes in this population. Prior validation work reported satisfactory reliability and construct validity, with test–retest reliability of 0.716–0.905, homogeneity reliability of 0.6501–0.8577, and split-half reliability of 0.6341–0.8400; correlations between the total score and subscales ranged from 0.7652 to 0.8726, and inter-subscale correlations ranged from 0.4027 to 0.7587 (Wang et al., 1997).

### 2.4 Training protocol

The exercise interventions for the HIFT and MICT groups were conducted over 8 weeks, with three 40-min sessions per week. The HIFT protocol integrated high-intensity intervals with gamified aerobic activities, specifically designed to target an intensity of 65%–88% HRmax for optimal health benefits while addressing adolescents’ preferences for varied and enjoyable exercise (Malik et al., 2019). The MICT protocol was based on two aerobic exercises and one fun exercise run, and the heart rate ranged from 65 to 75HRmax. The control group (CON) continued with their standard PE curriculum only. All training was delivered on an outdoor track and was led by two certified PE teachers, assisted by two supervisors who monitored technique and regulated intensity in real time. Each session included a 5-min structured warm-up comprising movements such as high knees, side lunges, arm circles, and light games, and ended with an identical 5-min cool-down in both intervention groups.

#### 2.4.1 HIFT protocol

The HIFT protocol consisted of three progressive stages:

Weeks 1–2: 30 s of work/30 s of rest (1:1), target intensity: 75%–80% HRmax.

Weeks 3–5: 30 s of work/20 s of rest (2:1.5), target intensity: 80%–85% HRmax.

Weeks 6–8: 40 s of work/20 s of rest (2:1), target intensity: 85%–88% HRmax.

Each exercise set was repeated twice, with a 3-min full rest between sets. For active recovery optimization, low-intensity dynamic activities (e.g., stepping and arm swinging) were performed to maintain the heart rate at 50%–60% HRmax during rest intervals. The HIFT training plan is shown in Supplementary Table S1. The weekly Friday extracurricular session introduced diversified activities. The main event was a 30-min timed circuit race structured as a team shuttle relay. Teams lined up at the starting point of an 80-m shuttle run course. Each of the three complete rounds consisted of three phases: a 4-min high-intensity exercise period, where participants sprinted the 80-m shuttle run one after another to the beat of high-tempo music, tagging the next teammate upon return and recording the total laps completed by the team; a 3-min designated rest period, where music stopped and all team members rested completely, either sitting or standing, to regulate breathing and ensure a total of 12 min of net high-intensity exercise while preventing excessive fatigue; and a 5-min active recovery phase, accompanied by calming music, during which the team performed low-intensity movements together—such as slow walking lunges, bodyweight squats, and stepping in place with deep breathing—to actively promote recovery while maintaining physical readiness for the next round. To emphasize the team-based and game-like characteristics of HIFT, students were organized into small groups of approximately 4–6 at each station. Within these groups, drills were delivered in cooperative or mildly competitive formats, such as partner “mirror” tasks and small-group repetition challenges completed within a fixed time.

#### 2.4.2 MICT protocol

The MICT protocol consisted of the following stages:

Weeks 1–2: Foundation rhythm: Simple steps and basic combinations that focused on following the rhythm and learning proper form (65%–70% HRmax).

Weeks 3–5: Tempo variation: Introduced slightly more complex moves and simple arm movements to the basic steps (65–75%HRmax).

Weeks 6–8: Integration and coordination: Incorporated fun skill advancement combinations and longer sequences that challenged coordination and memory while maintaining a continuous, steady pace (70–75%HRmax).

The Modified MICT protocol was designed as a continuous, rhythm-based aerobic circuit set to music. Each 30-min session consisted of three 10-min cycles. Every cycle included:

A 6-min aerobic block and a 4-min integrated strength and toning block that incorporated dynamic, bodyweight exercises performed at a brisk pace to blend muscular endurance with cardiovascular training. Thereby maintaining the target heart rate zone throughout (The MICT training plan is shown in Supplementary Table S2).

The weekly Friday extracurricular session was restructured to incorporate teamwork elements and task-oriented challenges into a 30-min fitness run. At designated checkpoints, teams collectively perform a prescribed number of repetitions of a light, dynamic exercise from the protocol (e.g., 20 tempo squats or 15 alternating lunges per person). The exercises are performed at a continuous pace before proceeding, ensuring the heart rate remains elevated and within the target zone throughout the activity.

### 2.5 Intervention implementation

Before the HIFT and MICT interventions, a specialized training module was implemented to ensure all participants mastered correct movement techniques and adhered to safety protocols. Exercise intensity was dually monitored via heart rate (HR) and the Borg Rating of Perceived Exertion (RPE) scale (6–20 points). All participants used HR monitors (Polar Team Pro, Polar Electro) to maintain intensity within the target range (75%–88% HRmax, phase-dependent) during work intervals and to guide active recovery. Given the latency in HR response during short high-intensity bouts, RPE served as the primary real-time reference for intensity adjustment, with an emphasis on safety and movement quality.

For the HIFT group, the target RPE range for high-intensity intervals was set at 16–18 (“Hard” to “Very Hard”), aligning with the 75%–88% HRmax intensity. If an RPE ≥16 was consistently reported, participants received cues to reduce pace or range of motion in subsequent sets to preserve technique and safety. RPE was recorded following the final repetition at each station. The MICT group targeted an RPE of 12–13 (“Somewhat Hard”), consistent with their prescribed HR zone. Sustained RPE ≥14 (“Hard”) prompted an immediate reduction in effort to prevent overexertion, with RPE assessed at 5-min intervals. Coaches provided real-time feedback to participants outside the target HR zone, using verbal cues (e.g., “Knees higher!”) to help regulate intensity.

To optimize engagement and spatial organization, a hexagonal dynamic training layout was used. At each station, students were organized into small groups of approximately 4–6 participants, which provided sufficient space for safe execution of movements and enabled teachers to observe and correct technique in real time. After completing each designated movement sequence, the participants moved clockwise to the next exercise station. To enhance participation and enjoyment, a two-tiered incentive mechanism was developed, consisting of immediate rewards and social reinforcement. Immediate rewards consisted of small, appealing tokens related to school or physical activity (e.g., sports wristbands or school-logo stationery). Each week, the school used the routine conduct score system to select the best-performing students in each class, and those who demonstrated better training engagement received additional conduct points. “Best performer” was defined using a simple rubric emphasizing observable effort, appropriate technique, and positive collaboration with peers, rather than maximal heart rate or absolute performance. This approach was adopted to promote a fair and mastery-oriented motivational climate and to avoid encouraging excessively intense exertion, while ensuring that exercise intensity was regulated through heart-rate monitoring and teacher feedback. Regarding immediate rewards, the best performer in each session received small prizes. Regarding social reinforcement, each session included an evaluation of group collaboration, and the top-performing team was granted special privileges. Specifically, the winning team in the HIFT group was allowed to choose the theme-appropriate background music for the next session, and the winning team in the MICT group was allowed to lead the exercise routine.

Compliance and fidelity were high: HR compliance rates reached 96% (HIFT) and 95.2% (MICT), with technical accuracy rates of 91.2% and 94.55%, respectively. Independent monitoring and behavioural observation confirmed an intervention completion rate of 98.7%, HR control deviation below 5%, and an average attendance rate of 96%.

### 2.6 Dietary intake

To minimize dietary-related confounding, participants maintained their usual school-based eating routine throughout the intervention and received no additional dietary counselling. Meals were mainly provided by the school canteen, with standardized menus for all students on the same day; detailed nutrient information (ingredients, recipes, and portion standards) was obtained from the school for dietary analysis. Dietary intake was assessed at baseline and immediately post-intervention using multiple-pass 24-h dietary recalls on 4 days (three nonconsecutive weekdays and one weekend day) (Wong et al., 2012). All recalls were conducted face-to-face by trained interviewers using standardized portion-size aids (food photographs and household utensils) to improve accuracy and consistency. Given the absence of an on-campus convenience store and the fixed canteen-based lunch/dinner schedule, the school setting provided a relatively controlled dietary environment, supporting the validity of the assessment. Total energy and macronutrient intakes (protein, fat, and carbohydrate) were estimated using the Boohee health software and its composition table (Boohee Info Technology Co., Shanghai, China) (Meng et al., 2022). This assessment aimed to confirm that no compensatory increases in energy or macronutrient intake occurred during the intervention, thereby strengthening the attribution of observed changes to exercise rather than dietary variation. Dietary data (mean ± SD) are reported in Supplementary Table S3.

### 2.7 Statistical analysis

All statistical analyses were performed with IBM SPSS Statistics (Version 26). Continuous data are expressed as mean ± standard deviation (SD). The normality of data distribution and homogeneity of variances were verified using the Shapiro–Wilk test and Levene’s test, respectively. Demographic variables, including age, height, weight, and BMI, were compared using one-way analysis of variance (ANOVA), while gender distribution was analyzed with the chi-square test. Age and gender were included as covariates where appropriate. To evaluate intervention effects, a 3 (group: HIFT, MICT, CON) × 2 (time: pre-test, post-test) repeated-measures ANOVA was employed. In cases of significant interaction or main effects, simple effects analysis with Bonferroni adjustment was conducted for *post hoc* comparisons. A p-value of less than 0.05 was considered statistically significant. For clarity in reporting results, within-group changes are presented as mean difference (Δ), calculated as post-intervention score minus baseline score.

## 3 Results

### 3.1 Effects of different exercise modalities on body composition

Significant group × time interactions were observed for key body-composition indicators. Overall, both exercise interventions reduced adiposity and central obesity relative to CON, with distinct profiles. MICT elicited the largest reduction in body fat percentage (Δ = −3.27%, p < 0.001), exceeding HIFT (Δ = −2.11%, p < 0.001), whereas CON showed no change (Δ = +0.57%, p = 0.208). Waist circumference decreased in both intervention groups (HIFT: Δ = −3.73 cm, p < 0.001; MICT: Δ = −3.29 cm, p < 0.001), while remaining stable in CON (Δ = +0.75 cm, p = 0.277). WHR improved similarly in both exercise groups (HIFT: Δ = −0.040, p < 0.001; MICT: Δ = −0.040, p < 0.001) but slightly worsened in CON (Δ = +0.027, p = 0.013). Both interventions reduced BMI (HIFT: Δ = −1.00 kg/m^2^; MICT: Δ = −1.29 kg/m^2^; both p < 0.001) and body weight (HIFT: Δ = −2.03 kg; MICT: Δ = −2.54 kg; both p < 0.001), whereas CON remained unchanged. Finally, muscle mass increased in both exercise groups (HIFT: Δ = +0.77 kg, p = 0.002; MICT: Δ = +0.51 kg, p = 0.037), with a greater gain in HIFT.

### 3.2 Effects of different exercise modalities on cardiorespiratory fitness and blood pressure parameters

A significant group × time interaction was found for $\dot{V}$ O_2_max. HIFT produced the greatest improvement in $\dot{V}$ O_2_max (Δ = +4.48 mL/kg/min, p < 0.001), compared with MICT (Δ = +2.05 mL/kg/min, p = 0.044) and CON (Δ = +0.42 mL/kg/min, p = 0.689), with a significant difference between HIFT and CON (p = 0.036). In the 1-min rope-jump test, only a time effect was observed; HIFT improved modestly (Δ = +3.71 times, p = 0.016) and MICT showed a non-significant trend (Δ = +2.76 times, p = 0.069). Systolic blood pressure decreased over time, with reductions in HIFT (Δ = −2.95 mmHg, p = 0.027) and MICT (Δ = −4.95 mmHg, p < 0.001), while CON showed no meaningful change. Diastolic blood pressure did not change significantly.

### 3.3 Effects of different exercise modalities on muscular fitness

Across muscular-fitness outcomes, improvements were mainly driven by time effects, with modality-specific advantages. Absolute handgrip strength increased significantly only in HIFT (Δ = +1.60 kg, p = 0.023). Relative strength (grip strength index) improved in both intervention groups (HIFT: Δ = +4.12%, p = 0.001; MICT: Δ = +3.86%, p = 0.001) but not in CON. Push-up performance improved in all groups, with larger gains in HIFT (Δ = +6.52 reps, p = 0.001) and MICT (Δ = +7.67 reps, p < 0.001) than in CON (Δ = +4.00 reps, p = 0.037). For lower-body explosive power, standing long jump improved substantially only in HIFT (Δ = +12.00 cm, p < 0.001), with no significant change in MICT (Δ = +4.95 cm, p = 0.117) or CON (Δ = +2.90 cm, p = 0.380).

### 3.4 Effects of different exercise modalities on speed and agility

A significant group × time interaction was observed for 50-m sprint (F (2,58) = 6.947, p = 0.002, η^2^ = 0.193). Both exercise groups demonstrated substantial enhancements in sprint speed (HIFT: Δ = −0.47 s, p < 0.001; MICT: Δ = −0.40 s, p < 0.001), while the control group showed minimal improvement (Δ = −0.05 s, p = 0.530). The HIFT group’s improvement was particularly notable, suggesting potential benefits for speed development through high-intensity functional training (Table 2).

**TABLE 2:** Changes in health-related physical fitness before and after intervention in the HIIT, MICT, and control Groups (M ± SD).

Variables	Group	Pretest M ± SD	Post-test M ± SD	Mean difference (95% CI)	p	Time (p)	Time × group (p)	Group (p)
Weight (kg)	HIFT	58.01 ± 8.26	55.98 ± 8.96	−2.03 [−2.65 to −1.21]***	0.000	0.000	0.640	0.099
	MICT	56.84 ± 7.84	54.30 ± 8.15#	−2.54 [-3.36 to −1.72]***	0.000			
	CON	61.41 ± 6.36	61.28 ± 6.39	−0.13 [-0.99 to 0.73]	0.761			
BF (%)	HIFT	29.94 ± 4.37	27.82 ± 3.93	−2.11 [−2.96 to −1.27]***	0.000	0.000	0.000	0.848
	MICT	30.25 ± 3.82	26.98 ± 4.50	−3.27 [-4.12 to −2.42]***	0.000			
	CON	27.83 ± 4.87	28.40 ± 4.67	0.57 [−0.3 to 1.46]	0.208			
WC (cm)	HIFT	77.85 ± 6.00	74.12 ± 4.90#	−3.73 [−5.03 to −2.43]***	0.000	0.000	0.000	0.162
	MICT	76.61 ± 5.60	73.33 ± 4.74#	−3.29 [-4.58 to −1.99]***	0.000			
	CON	77.85 ± 5.02	78.60 ± 7.08	0.75 [−0.62 to 2.11]	0.277			
WHR	HIFT	0.86 ± 0.03	0.82 ± 0.03#	−0.04 [-0.06 to −0.02]***	0.000	0.004	0.000	0.283
	MICT	0.87 ± 0.06	0.83 ± 0.045#	−0.04 [-0.06 to −0.02]***	0.000			
	CON	0.85 ± 0.05	0.88 ± 0.10	0.03 [0.01 to 0.05]*	0.013			
Muscle content (kg)	HIFT	21.45 ± 4.10	22.22 ± 4.08	0.77 [0.30 to 1.24]**	0.002	0.035	0.004	0.543
	MICT	20.29 ± 4.27	20.70 ± 4.07	0.51 [−0.03 to 0.98]*	0.037			
	CON	21.74 ± 3.72	21.36 ± 3.61	0.38 [−0.88 to 0.12]	0.132			
BMI (kg/m^2^)	HIFT	23.76 ± 2.05	22.76 ± 2.43	−1.00 [−1.35 to −0.65]***	0.000	0.000	0.000	0.09
	MICT	23.00 ± 1.59	21.69 ± 1.83#	−1.29 [−1.63 to −0.94]***	0.000			
	CON	23.72 ± 1.90	23.60 ± 1.91	−0.12 [−0.49 to 0.25]	0.510			
$\dot{V}$ O_2_max (mL/kg/min)	HIFT	40.10 ± 6.19	44.57 ± 5.37	4.48 [2.49 to 6.47]***	0.000	0.000	0.023	0.300
	MICT	40.05 ± 5.757	42.09 ± 6.30	2.05 [0.06 to 4.04]*	0.044			
	CON	39.68 ± 3.86	40.10 ± 4.37	0.42 [−1.67 to 2.51]	0.689			
1-min rope jump (times)	HIFT	158.48 ± 36.61	162.19 ± 33.12	3.71 [0.73 to 6.70]*	0.016	0.007	0.41	0.996
	MICT	158.19 ± 28.23	160.95 ± 26.84	2.76 [-0.22 to 5.75]	0.069			
	CON	159.42 ± 15.40	160.26 ± 14.72	0.84 [−2.30 to 3.98]	0.593			
Systolic blood pressure (mmHg)	HIFT	113.10 ± 8.63	110.14 ± 8.28	−2.95 [−5.55 to 0.35]*	0.027	0.000	0.213	0.357
	MICT	112.76 ± 7.16	107.81 ± 7.06	−4.95 [−7.55 to −2.35]***	0.000			
	CON	115.79 ± 16.41	114.16 ± 14.99	−1.63 [−4.37 to 1.10]	0.237			
Diastolic blood pressure (mmHg)	HIFT	66.62 ± 9.12	64.95 ± 6.10	−1.67 [−4.96 to 1.62]	0.315	0.334	0.417	0.448
	MICT	66.62 ± 7.90	64.57 ± 8.54	−2.05 [−5.36 to 1.24]	0.218			
	CON	67.32 ± 3.76	68.21 ± 4.11	0.90 [−2.56 to 4.35]	0.600			
Standing long jump (cm)	HIFT	154.71 ± 24.60	166.71 ± 28.65	12.00 [5.78 to 23.16]***	0.000	0.001	0.111	0.885
	MICT	159.14 ± 25.45	164.10 ± 23.64	4.95 [−1.27 to 11.18]	0.117			
	CON	162.68 ± 18.61	165.46 ± 17.63	2.90 [-3.65 to 9.44]	0.380			
Handgrip strength (kg)	HIFT	18.34 ± 4.91	19.93 ± 6.19	1.60 [0.23 to 2.97]*	0.023	0.005	0.651	0.713
	MICT	17.88 ± 4.51	19.14 ± 4.77	1.26 [−0.11 to 2.63]	0.070			
	CON	19.63 ± 5.90	20.32 ± 8.27	0.68 [−0.76 to 2.12]	0.345			
Grip strength index (%)	HIFT	31.73 ± 7.90	35.84 ± 9.89	4.12 [1.84 to 6.40]**	0.001	0.000	0.106	0.886
	MICT	31.75 ± 7.86	35.60 ± 8.78	3.86 [1.58 to 6.14]**	0.001			
	CON	32.05 ± 9.42	32.93 ± 12.67	0.88 [−1.52 to 3.28]	0.466			
Push-ups (reps)	HIFT	16.86 ± 13.92	23.38 ± 15.68	6.52 [2.96 to 10.09]**	0.001	0.000	0.359	0.740
	MICT	17.95 ± 14.01	25.62 ± 17.72	7.67 [4.10 to 11.23]***	0.000			
	CON	16.42 ± 10.55	20.42 ± 11.91	4.00 [0.25 to 7.75]*	0.037			
50 m(s)	HIFT	9.06 ± 0.82	8.59 ± 0.68	−0.47 [−0.64 to −0.31]***	0.000	0.000	0.002	0.431
	MICT	9.39 ± 1.08	8.99 ± 0.963	−0.40 [−0.56 to −0.23]***	0.000			
	CON	9.09 ± 1.01	9.03 ± 1.01	−0.05 [−0.23 to 0.12]	0.530			

SD, standard deviation; CI: confidence interval; HIFT, high-intensity functional training; MICT, moderate-intensity continuous training; CON, control training; BF%: body fat percentage; WC, waist circumference; WHR, waist-to-hip ratio; BMI: body mass index; $\dot{V}$ O_2_max, maximal oxygen uptake; reps, repetitions; s, seconds. All analyses controlled for gender and age. * indicates within-group significance (*p < 0.05; **p < 0.01; ***p < 0.001); # denotes comparisons with the control group (#p < 0.05; ##p < 0.01; ###p < 0.001).

### 3.5 Effects of different exercise modalities on emotional modulation

Repeated-measures ANOVA showed significant main effects of time for all mood dimensions (all p < 0.01). Significant group × time interactions were also found for anger (F (2,58) = 3.888, p = 0.026, ηp^2^ = 0.118), fatigue (F (2,58) = 3.111, p = 0.050, ηp^2^ = 0.097), depression (F (2,58) = 5.580, p = 0.006, ηp^2^ = 0.161), self-esteem (F (2,58) = 14.566, p < 0.001, ηp^2^ = 0.334), total negative mood (F (2,58) = 8.720, p < 0.001, ηp^2^ = 0.231), total positive mood (F (2,58) = 15.345, p < 0.001, ηp^2^ = 0.346), and total mood disturbance (TMD; F (2,58) = 17.180, p < 0.001, ηp^2^ = 0.372).

Regarding negative affect, MICT produced the most consistent improvements. Anger decreased most in MICT (Δ = −2.429 points, p < 0.001), exceeding CON (p < 0.05), and fatigue was reduced only in MICT (Δ = −1.143, p < 0.001). Depression improved in both intervention groups (HIFT: Δ = −1.524 points; MICT: Δ = −1.810 points; both p < 0.001), with changes greater than those in CON. Consistently, total negative mood declined most in MICT (Δ = −8.571 points, p < 0.001), followed by HIFT (Δ = −6.381 points, p < 0.001) and CON (Δ = −4.158 points, p < 0.001).

**FIGURE 2:**
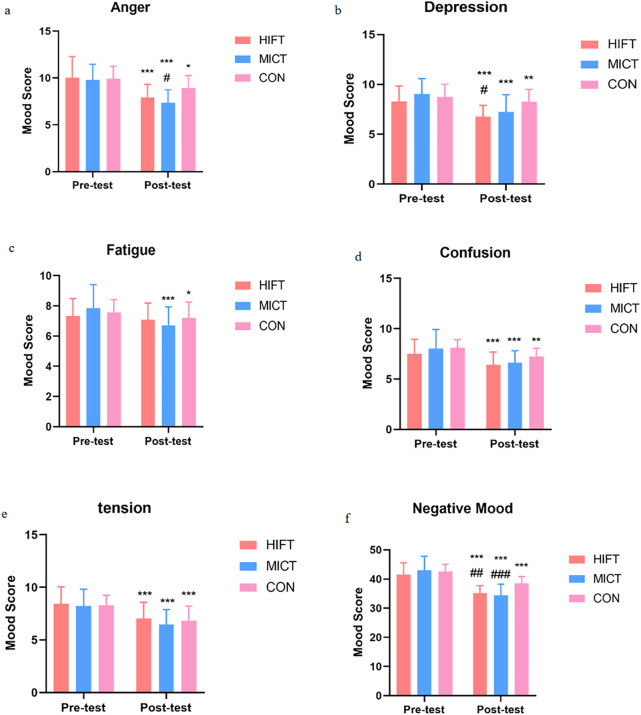
Changes in negative emotional states across the HIFT, MICT, and CON. HIFT: high-intensity functional training; MICT: moderate-intensity continuous training; CON: control training. **(a)** Changes in anger scores across the groups. **(b)** Changes in depression scores across the groups. **(c)** Changes in fatigue scores across the groups. **(d)** Changes in confusion scores across the groups. **(e)** Changes in tension scores across the groups. **(f)** Changes in negative mood scores across the groups. * indicates within-group significance (*p < 0.05; **p < 0.01; ***p < 0.001); # denotes com-parisons with the control group (#p < 0.05; ##p < 0.01; ###p < 0.001).

For tension and confusion, only time effects were observed (tension: F (1,58) = 107.867, p < 0.001; confusion: F (1,58) = 42.130, p < 0.001), indicating general improvements across groups without between-group differences (Figure 2).

The HIFT intervention demonstrated superior efficacy in enhancing positive emotional states. Specifically, the HIFT group showed a greater increase in self-esteem (Δ = +3.238, p < 0.001) compared to both the MICT group (Δ = +2.238 points, p < 0.001) and the control group, with the difference reaching statistical significance (p < 0.001). Similarly, HIFT led to a significantly greater improvement in vigor (Δ = +2.429 points, p < 0.001) relative to the control group (Δ = +1.263 points, p < 0.01). Consequently, the HIFT group also exhibited the largest gains in total positive mood (Δ = +5.667, p < 0.001), significantly outperforming both the MICT (Δ = +3.952 points, p < 0.001) and control groups (Δ = +2.158 points, p < 0.001). These findings suggest that HIFT may possess unique potential for rapidly enhancing feelings of energy and self-worth.

**FIGURE 3:**
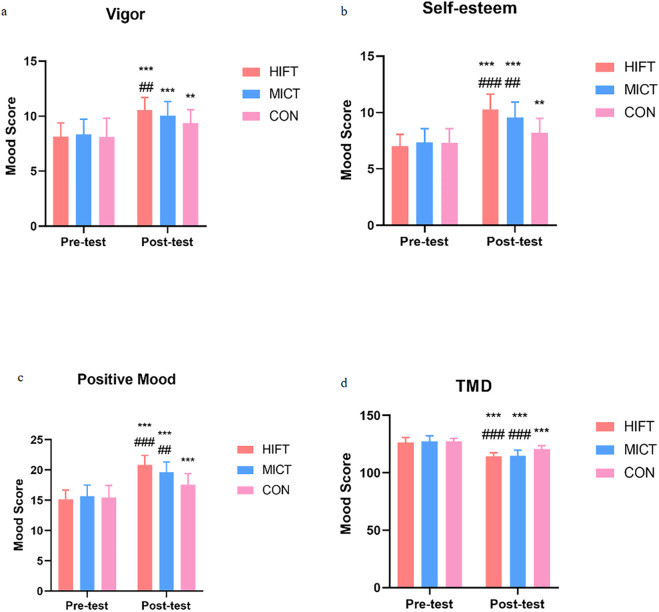
Changes in positive emotional and total mood disturbance states across the HIFT, MICT, and CON. HIFT: high-intensity functional training; MICT: moderate-intensity continuous training; CON: control training. **(a)** Changes in vigor scores across the groups. **(b)** Changes in self-esteem scores across groups. **(c)** Changes in positive mood scores across the groups. **(d)** Changes in total mood disturbance scores across the groups.* indicates within-group significance (*p < 0.05; **p < 0.01; ***p < 0.001); # denotes comparisons with the control group (#p < 0.05; ##p < 0.01; ###p < 0.001).

Both interventions yielded substantial improvements in global emotional health. The TMD score decreased by 11.952 points in the HIFT group (p < 0.001) and by 12.524 points in the MICT group (p < 0.001), with both reductions being significantly greater than that in the control group (Δ = −6.316 points, p < 0.001). These results confirm that both HIFT and MICT are highly effective means of improving overall emotional health in adolescents (Figure 3).

### 3.6 Effects of different exercise modalities on psychological health

A significant group × time interaction was observed for anxiety (F (2,58) = 3.704, p = 0.031, ηp^2^ = 0.113), emotional imbalance (F (2,58) = 5.149, p = 0.009, ηp^2^ = 0.149), and total mental health score (F (2,58) = 6.183, p = 0.004, ηp^2^ = 0.175), with a marginal interaction for interpersonal sensitivity (F (2,58) = 3.153, p = 0.050, ηp^2^ = 0.098).

MICT elicited the largest reductions in anxiety (Δ = −0.586 points, p < 0.001) and depressive symptoms (Δ = −0.429 points, p < 0.001), both significantly greater than CON (both p < 0.01), and also produced the strongest improvement in emotional imbalance (Δ = −0.595 points, p < 0.001; vs. CON p < 0.01). HIFT also reduced anxiety (Δ = −0.286 points, p = 0.002), depressive symptoms (Δ = −0.229 points, p = 0.027), and emotional imbalance (Δ = −0.486 points, p < 0.001), but the latter did not differ significantly from CON.

**FIGURE 4:**
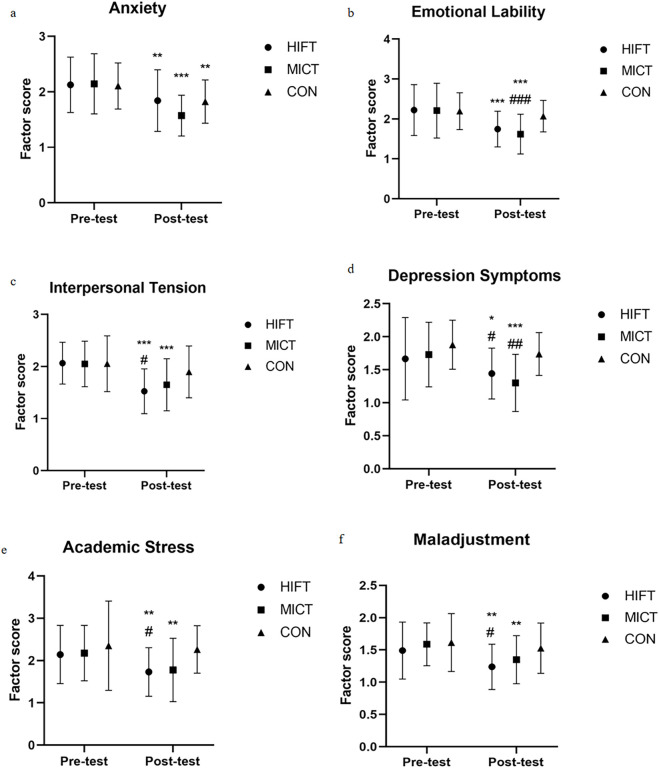
Changes in Mental Health (Part 1) states across the HIFT, MICT, and CON. HIFT: high-intensity functional training; MICT: moderate-intensity continuous training; CON: control training. **(a)** Changes in anxiety scores across the groups. **(b)** Changes in emotional lability scores across groups. **(c)** Changes in interpersonal tension scores across the groups. **(d)** Changes in depression symptom scores across the groups. **(e)** Changes in academic stress scores across the groups. **(f)** Changes in maladjustment scores across the groups. * indicates within-group significance (*p < 0.05; **p < 0.01; ***p < 0.001); # denotes comparisons with the control group (#p < 0.05; ##p < 0.01; ###p < 0.001).

For interpersonal sensitivity, HIFT showed the greatest improvement (Δ = −0.538 points, p < 0.001), exceeding CON (p < 0.05), whereas MICT also improved (Δ = −0.419 points, p < 0.001) without a significant between-group difference versus CON. HIFT significantly alleviating academic stress (Δ = −0.410 points, p = 0.007; vs. CON p < 0.05) and maladjustment (Δ = −0.243 points, p = 0.002; vs. CON p < 0.05). Although MICT improved academic stress (Δ = −0.400 points, p = 0.009) and maladjustment (Δ = −0.248 points, p = 0.001), these changes were not significantly different from CON (Figure 4).

For other dimensions, including obsessive-compulsive symptoms, paranoid ideation, and psychological imbalance, significant main effects of time were observed (obsessive-compulsive: F (1,58) = 8.678, p = 0.005; paranoid ideation: F (1,58) = 39.093, p < 0.001; psychological imbalance: F (1,58) = 12.49, p = 0.001), indicating improvements across all groups over time, with no significant between-group differences. For hostility, although both HIFT and MICT groups showed a trend toward improvement, the changes did not reach statistical significance (main effect of time: p = 0.1).

**FIGURE 5:**
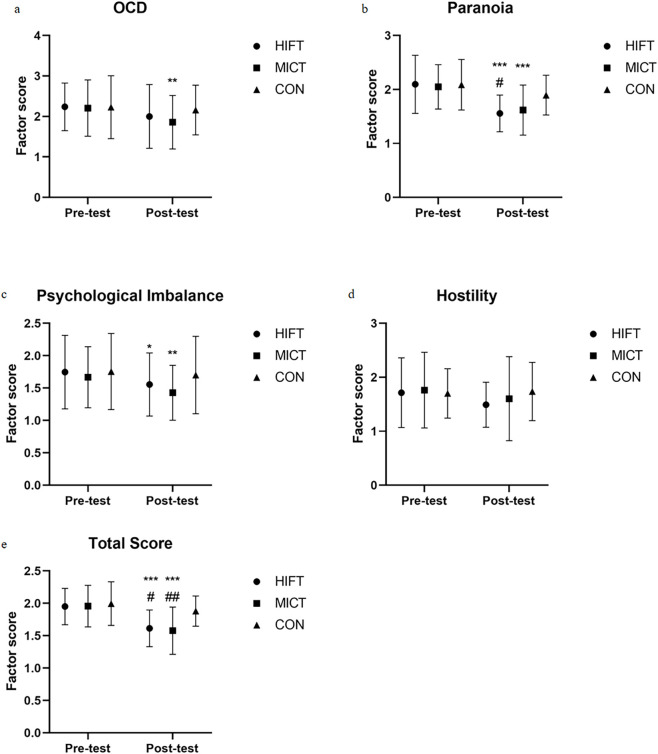
Changes in Mental Health (Part 2) states across the HIFT, MICT, and CON. HIFT: high-intensity functional training; MICT: moderate-intensity continuous training; CON: control training. **(a)** Changes in obsessive-compulsive disorder scores across the groups. **(b)** Changes in paranoia scores across groups. **(c)** Changes in psychological imbalance scores across the groups. **(d)** Changes in hostility scores across the groups. **(e)** Changes in Total Score scores across the groups * indicates within-group significance (*p < 0.05; **p < 0.01; ***p < 0.001); # denotes comparisons with the control group (#p < 0.05; ##p < 0.01; ###p < 0.001).

Regarding overall mental health, both intervention groups exhibited significant improvements. A significant time-by-group interaction was found for the total mental health score (F (2,58) = 6.183, p = 0.004, ηp^2^ = 0.175). Post-hoc analyses revealed that the HIFT group showed a marked reduction (Δ = −0.333 points, p < 0.001), as did the MICT group (Δ = −0.386 points, p < 0.001), with both decreases being significantly greater than that observed in the control group (Δ = −0.111 points, p = 0.071) (Figure 5).

## 4 Discussion

This study examined the differential effects of HIFT and MICT on body composition, physical fitness, emotional states, and psychological health in overweight/obese adolescents. Both intervention modalities produced broad and meaningful health benefits, though their strengths varied across physical and psychological domains. Importantly, dietary intake monitoring suggests that these benefits were achieved without systematic dietary changes, aligning with lifestyle intervention aims in school settings.

Both exercise modalities effectively reduced adiposity and central obesity, which is consistent with prior evidence supporting school-based exercise interventions for adolescent obesity management (Dias et al., 2018; Meng et al., 2022). Notably, the two interventions showed differentiated response profiles. HIFT produced a pronounced reduction in waist circumference alongside an increase in muscle mass, a pattern that aligns with reports that higher-intensity interval/functional approaches may preferentially improve abdominal adiposity and body recomposition (Racil et al., 2013; Weston et al., 2016). Mechanistically, the combination of high metabolic stress and multi-joint functional movements in HIFT may elevate post-exercise oxygen consumption and prolong lipid oxidation (Panissa et al., 2021), while also increasing resting metabolic rate and improving vascular function in youth with obesity (Chuensiri et al., 2018). In addition, the resistance-like components embedded in functional circuits may stimulate muscle protein synthesis and support lean mass gains, thereby facilitating favorable body recomposition (Ben-Zeev and Okun, 2021; Silva-Reis et al., 2022). In contrast, MICT elicited a larger reduction in overall body fat percentage and body weight, plausibly reflecting sustained energy expenditure and a stable caloric deficit mechanism typical of continuous aerobic training (Kessler et al., 2012). Although both groups improved waist-to-hip ratio (WHR), the similar WHR reduction may reflect divergent pathways: HIFT may reduce abdominal fat while preserving or enhancing hip/gluteal musculature, whereas MICT-related hip changes may primarily reflect fat loss (Osawa et al., 2014). Clinically, the observed WHR improvement is relevant because WHR is closely linked to visceral adiposity–related metabolic risk and cardiometabolic outcomes (Xie et al., 2024), and the control group’s unfavorable adiposity trend further underscores the importance of structured exercise for preventing obesity progression.

Regarding blood pressure regulation, MICT demonstrated superior efficacy with significantly greater systolic blood pressure reduction versus HIFT. This aligns with established evidence that aerobic exercise lowers blood pressure through vascular endothelial improvement and sympathetic nervous system modulation (Alberga et al., 2016; Chen et al., 2016). In terms of improving the cardiovascular and metabolic health, as well as weight management of overweight/obese adolescents, some studies have shown that HIFT may be more effective than MICT. However, it is worth noting that this study contradicts the results of these studies (Garcia-Hermoso et al., 2016; Meng et al., 2022). Given that ≥4 mmHg systolic reduction decreases cardiovascular mortality by 5%–20% (Taylor et al., 2011), MICT offers valuable non-pharmacological management for obesity-related hypertension risk.

Cardiorespiratory fitness is a critical target because adolescents with overweight/obesity typically show reduced aerobic fitness, which elevates future cardiovascular disease risk (Suriano et al., 2010). In this trial, HIFT produced a markedly greater increase in $\dot{V}$ O_2_max than MICT, consistent with school-based high-intensity exercise literature showing robust improvements in aerobic capacity among youth (Garcia-Hermoso et al., 2016; Bogataj et al., 2021; Meng et al., 2022). The underlying mechanism likely relates to the stronger stimulus for central and peripheral aerobic adaptations induced by repeated high-intensity bouts, as supported by mechanistic and synthesis evidence in high-intensity multimodal/interval training research (Claudino et al., 2018; Duncombe et al., 2022). Moreover, HIFT may enhance motor unit recruitment and contraction characteristics, potentially contributing to broad functional performance gains (Heinrich et al., 2021).

Consistent with the principle of training specificity, HIFT demonstrated clearer advantages in lower-body power and speed-related outcomes, including improvements in standing long jump and sprint performance. Similar benefits have been observed in functional high-intensity circuit training, which can improve body composition, aerobic fitness, and strength parameters in populations with overweight/obesity (Sperlich et al., 2017). These performance gains likely reflect explosive movement patterns that recruit fast-twitch fibers and improve neuromuscular coordination, a commonly proposed pathway for speed/power adaptation in youth-oriented high-intensity training (Paulino da Silva Bento et al., 2021; Bossmann et al., 2022). HIFT’s integrated benefits were further evidenced by exclusive improvement in 1-min rope jump. At the same time, both HIFT and MICT improved several strength/endurance indicators, suggesting that either modality can enhance functional fitness in school programs, albeit with different emphases.

A key finding of this study is that HIFT and MICT yielded distinct yet complementary psychological benefits. MICT appeared particularly effective for reducing negative mood and internalizing symptoms (e.g., anger, fatigue, depressive mood, anxiety), consistent with evidence that moderate-intensity aerobic activity can attenuate negative emotional responses and stress-related reactivity (Long et al., 2021). This pattern may be explained by the rhythmic and steady-state nature of MICT, which is often associated with improved autonomic regulation, enhanced vagal tone, and increased availability of mood-regulating neurotransmitters and neuroendocrine mediators (Foley and Fleshner, 2008; Xie et al., 2021; Yao et al., 2021). In contrast, HIFT more strongly enhanced positive mood dimensions such as vigor and self-esteem and showed additional benefits for interpersonal sensitivity, consistent with school-based high-intensity functional programs improving psychophysical wellbeing in adolescents (Cataldi et al., 2021). From a neurobiological perspective, high-intensity exercise may activate reward-related pathways and elevate neurotrophic factors such as BDNF, supporting motivational and affective improvements (Murawska-Cialowicz et al., 2021). Similar improvements in adolescent self-esteem following high-intensity exercise have also been reported (Li and Zhou, 2025). In addition, by incorporating both high-intensity functional movements and team-based elements, HIFT offers a unique pathway to support not only physical wellbeing but also emotional wellbeing in adolescents (Koon et al., 2023).

Beyond modality-specific effects, our findings support an integrated physical–psychological pathway in adolescents with overweight/obesity. Existing evidence indicates that higher muscular fitness is associated with more favorable psychological functioning and self-related constructs, suggesting that physical competence can translate into improved self-esteem and wellbeing (Jorgensen et al., 2017).

Improvements in cardiorespiratory fitness, muscular performance, and BMI may also enhance perceived behavioral control and self-efficacy, which in turn supports better emotional regulation and engagement (Haung et al., 2014). This is particularly relevant because overweight/obesity is frequently linked to body image dissatisfaction, lower self-esteem, and heightened anxiety vulnerability in youth (He et al., 2014; Haghighi et al., 2016). Consistent with this framework, muscular fitness has been associated with psychological positive health and reduced health complaints in children and adolescents (Padilla-Moledo et al., 2012; Jeoung et al., 2013). The experience of successfully completing challenging physical tasks may further foster resilience, which is associated with better quality of life and mental health status (Perez-Cruzado et al., 2018) and is linked to lower depression risk and reduced anxiety/stress vulnerability (Wang et al., 2014; Zhao-Hui, 2020). Adolescence is also a sensitive period during which body image concerns and weight-related stigma can heighten social anxiety and exposure to bullying, potentially worsening mental health trajectories (Chang and Gable, 2013; Liu et al., 2020). In this context, evidence suggests that weight management interventions can mitigate depressive symptoms and potentially disrupt long-term risk pathways (Sarmiento-Riveros et al., 2025). Furthermore, structured group exercise can provide social reinforcement and peer-related support that may buffer psychological distress (Andersen et al., 2019), which may partially explain the broad improvements observed in both intervention groups.

Taken together, the divergent benefits observed here likely reflect the inherent “exercise dose” characteristics of each modality. HIFT’s short, high-intensity bouts may preferentially drive neuromuscular and aerobic adaptations while acutely enhancing positive affect through arousal and mastery experiences, whereas rhythm-based MICT may provide a more stable psychophysiological context that supports sustained fat oxidation and reductions in negative affect. Practically, these findings support a more precision-oriented selection of school-feasible exercise modalities depending on prioritized physical versus psychological targets.

This study was implemented during the post-pandemic recovery period, when adolescents experienced reduced physical activity, heightened emotional volatility, and increased obesity risk. In such a context, structured group exercise may offer dual benefits by improving physical fitness while supporting emotional resilience and social functioning. A further strength of this trial is the relatively standardized school food environment, and dietary monitoring confirmed no systematic dietary changes, supporting an energy-balance–controlled, psychologically safe, and stigma-sensitive school intervention model targeting both physical and mental dimensions of adolescent obesity.

Several limitations should be noted. First, participants were recruited from a single school and limited to adolescents aged 11–12 years with overweight/obesity, which may restrict generalizability. Second, although dietary intake was assessed using repeated 24-h recalls, recall-based methods may not fully capture out-of-school consumption and day-to-day variability. Third, habitual physical activity outside the intervention sessions was not objectively quantified, which may have introduced residual confounding. Fourth, pubertal stage was not assessed, which may influence responsiveness to training. Finally, the intervention duration was relatively short; longer follow-up and mechanistic measures (e.g., biomarkers of metabolic/inflammatory pathways) are needed to clarify the persistence and biological basis of modality-specific benefits.

## 5 Conclusion

This study demonstrates that HIFT and MICT effectively improved the health status of overweight/obese adolescents through distinct pathways. HIFT yielded superior outcomes in reducing waist circumference, enhancing lower-body explosive power (as assessed by standing long jump), and promoting positive psychological traits, whereas MICT proved more effective in overall fat reduction and emotional regulation. The complementary benefits of these two models underscore the value of implementing tailored exercise prescriptions in school-based interventions to address the complex health needs of overweight and obese adolescents.

## Data Availability

The original contributions presented in the study are included in the article/Supplementary Material, further inquiries can be directed to the corresponding authors.
