# Supplementary material for: Divergent effects of high-intensity functional training and moderate-intensity continuous training in adolescents with overweight/obesity: a randomized controlled trial on body composition, physical fitness, and psychological health
Source: Front Physiol. 2026 Mar 16;17:1756285. doi: 10.3389/fphys.2026.1756285 (PMC13033501; doi:10.3389/fphys.2026.1756285)
Supplement: Supplementary file 1 [file Table1.docx]

**Table S1.** HIFT Training Program.

Monday: Foundational Strength & Stability Day

Theme: Focus on establishing proper movement patterns and core stability

| **Week** | **Work/Rest** | **Sample Exercise Sets (Each performed twice per session)** |
| --- | --- | --- |
| 1-2 | 30s/30s | Set 1: Marching in Place - Wall Sit - High Plank  Set 2: Desk Push-Ups - Glute Bridges - Bird-Dog – Crunches  Set 3: Bodyweight Squats - Calf Raises (In Place) - Farmer's Walk (with Water Bottles) - Standing Knee Raises |
| 3-5 | 30s/20s | Set 1: Jumping Jacks - Sumo Squats - Plank  Set 2: Knee Push-Ups - Single-Leg Glute Bridges (Alternating) - Side Plank (Modified) - Hip Rotations (Left and Right)  Set 3: Lunge Jumps (Slow Pace) - Bent-Over Rows (Bodyweight) - Dead Bugs - Lunge Balance with Knee Lift |
| 6-8 | 40s/20s | Set 1: Quick Shuffles - Squat Jumps (No Off-The-Ground) - Arm-Leg Coordination (Standing Alternating Foot Touches)  Set 2: Burpees with Overhead Reach (No Jump) - Traveling Mountain Climbers - Russian Twists - Traveling Mountain Climbers  Set 3: Skater Steps (Side Jumps, Low Impact) - Spider Lunges - Supine Crunches with Knee Taps - Side Shuffles with Floor Touches |

Wednesday: Coordination & Integration Training Day

Theme: Introduce more multi-planar movements and coordination challenges

| **Week** | **Work/Rest** | **Sample Exercise Sets (Each performed twice per session)** |
| --- | --- | --- |
| 1-2 | 30s/30s | Set 1: Jumping Jacks (Slow Pace) - Shoulder-Tap Plank - Heel Taps (Front & Side)  Set 2: Side Lunges (Shallow Depth) - Standing Alternating Knee Kicks - Cross Punches - March in Place  Set 3: "V" Steps - Modified Burpees - Diagonal Crunches - Standing Side Bends |
| 3-5 | 30s/20s | Set 1: High Knees in Place - Reverse Lunges - Walking Plank (Side to Side)  Set 2: Grapevine Steps - Uppercuts - Butt Kicks - Cross Uppercuts  Set 3: Side Shuffles with Floor Touches - Bicycle Crunches - Wall Sit Pulses - Side Lunge Balance |
| 6-8 | 40s/20s | Set 1: Quick Shuffles - Squat Jumps (No Off-The-Ground) - Arm-Leg Coordination (Standing Alternating Foot Touches)  Set 2: Burpees with Overhead Reach (No Jump) - Traveling Mountain Climbers - Russian Twists - Traveling Mountain Climbers  Set 3: Skater Steps (Side Jumps, Low Impact) - Spider Lunges - Supine Crunches with Knee Taps - Side Shuffles with Floor Touches |
