# Supplementary material for: Divergent effects of high-intensity functional training and moderate-intensity continuous training in adolescents with overweight/obesity: a randomized controlled trial on body composition, physical fitness, and psychological health
Source: Front Physiol. 2026 Mar 16;17:1756285. doi: 10.3389/fphys.2026.1756285 (PMC13033501; doi:10.3389/fphys.2026.1756285)
Supplement: Supplementary file 2 [file Table2.docx]

**Table S2.** MICT Training Program.

| **Week** | **Stage & Target Intensity** | **Sample 10-Minute Cycle Structure (Repeated 3 times per session)** |
| --- | --- | --- |
| 1-2 | Foundation Rhythm 65-70% HRmax | A. 6-min Aerobic Block:  March & Reach (March in Place + Alternating Arm Raises)  Step Touch + Bicep Curl (Side Step Touches + Bicep Curls)  Heel Digs + Shoulder Press (Heel Digs + Shoulder Presses)  Knee Lift + Overhead Clap (Knee Raises + Overhead Claps)  Single Arm Row March (March in Place + Alternating Single-Arm Rows)  B. 4-min Integrated Strength & Toning Block:  Tempo Goblet Squats: Clasp your hands in front of your chest, lower slowly (3 seconds) and rise quickly (1 second).  Standing Cross Crunches: Lift your knee while touching it with your elbow, emphasizing trunk rotation and balance.  Incline Push-ups: Perform against a wall or table edge, and control the tempo.  Glute Bridge Marches: Train glute and core stability. |
| 3-5 | Tempo Variation 65-75% HRmax | A. 6-min Aerobic Block:  Double Grapevine: Step right-left-right, then lift one leg. Repeat in the opposite direction, and add arm circles.  Box Step + Punch: Step forward-side-back-side, coordinate with alternating punches, and integrate combat elements.  V-Step + Jazz Hands: Spread your hands when stepping out to enhance expressiveness.  Mambo Step + Hip Sway: Take forward-back rocking steps, add hip rhythm, and improve coordination.  Jack U: When jumping your feet outward, move your arms from both sides in a "U" shape to meet in front of your body.  B. 4-min Integrated Strength & Toning Block:  Lunge & Twist: Step back into a lunge, and at the same time rotate your body toward the lunging leg to activate the core.  Sumo Squat with Uppercut: Throw an uppercut when squatting down, and retract your fist when standing up to integrate the explosive power of the lower and upper body.  Bird-Dog: Get into a four-point support position, extend the opposite arm and leg, and train core anti-rotation and balance to the maximum.  Calf Raise with Lateral Raise: Train the calves and shoulders to improve posture. |
| 6-8 | Integration & Coordination 70-75% HRmax | 6-min Aerobic Block:  Step-Kick-Curl-Press: A smooth combination that integrates multi-joint movements, which is highly challenging.  Cha-Cha Cha + Shoulder Roll: Step-step-together, with the addition of isolated shoulder rolls to enhance the sense of dance.  Shuffle-Ball-Change: A classic dance step that is quick and light, used to train footwork.  Traveling Step Touch with Arm Waves: Move to the side while performing continuous wave movements with the arms.  Rocking Kickbacks: Rock the body back and forth, coordinating with backward hooked legs, full of dynamism.  4-min Integrated Strength & Toning Block:  Squat into Curtsy Lunge: A dynamic lower-body combination that trains multi-planar movement ability.  Plank with Alternating Knee to Elbow: A dynamic core exercise that challenges coordination and core endurance.  Renegade Rows: Perform alternating rowing movements (bodyweight optional) while in a high plank position to train full-body tension control.  Lateral Lunge with Windmill Touch: During a lateral lunge, touch the ground with the opposite hand and extend the other arm upward, significantly improving flexibility and chain-based force generation. |
