# Supplementary material for: Divergent effects of high-intensity functional training and moderate-intensity continuous training in adolescents with overweight/obesity: a randomized controlled trial on body composition, physical fitness, and psychological health
Source: Front Physiol. 2026 Mar 16;17:1756285. doi: 10.3389/fphys.2026.1756285 (PMC13033501; doi:10.3389/fphys.2026.1756285)
Supplement: Supplementary file 3 [file Table3.docx]

**Table S3.** Daily energy and nutrient intake at baseline and post-intervention by group (mean ± SD).

| **Nutrient** | **HIFT Pre** | **HIFT Post** | **MICT Pre** | **MICT Post** | **CON Pre** | **CON Post** | **G×T p** |
| --- | --- | --- | --- | --- | --- | --- | --- |
| Energy (kcal/day) | 2035±69 | 2056±69 | 2041±50 | 2052±54 | 2034±64 | 2046±82 | 0.245 |
| Protein (g/day) | 71.8±1.9 | 72.6±2.5 | 71.9±2.1 | 72.8±2.5 | 71.6±1.6 | 72.5±2.5 | 0.997 |
| Carbohydrate (g/day) | 272.9±7.6 | 274.5±7.2 | 276.1±6.8 | 276.6±7.1 | 271.8±7.0 | 272.5±7.5 | 0.627 |
| Fat (g/day) | 73.2±3.9 | 74.1±3.0 | 72.7±2.7 | 73.5±2.7 | 72.3±2.7 | 73.4±4.4 | 0.974 |
| Dietary fiber (g/day) | 18.6±2.5 | 19.1±2.8 | 18.2±2.4 | 18.8±2.7 | 18.4±2.3 | 18.4±3.9 | 0.785 |

Note: Data are presented as mean±standard deviation, based on 24-hour dietary recall assessment. The group×time interaction effects for all nutrients were not significant (p > 0.05), indicating that the regular school diet remained stable during the intervention period.
